# The Relationships among “STAY-GREEN” Trait, Post-Anthesis Assimilate Remobilization, and Grain Yield in Rice (*Oryza sativa* L.)

**DOI:** 10.3390/ijms232213668

**Published:** 2022-11-08

**Authors:** Yuguang Zang, Yijia Yao, Zheshu Xu, Baoqing Wang, Yiqi Mao, Weilu Wang, Weiyang Zhang, Hao Zhang, Lijun Liu, Zhiqin Wang, Guohua Liang, Jianchang Yang, Yong Zhou, Junfei Gu

**Affiliations:** 1 Jiangsu Key Laboratory of Crop Genetics and Physiology, Jiangsu Key Laboratory of Crop Cultivation and Physiology, Agricultural College, Yangzhou University, Yangzhou 225009, China; 2 Jiangsu Co-Innovation Center for Modern Production Technology of Grain Crops, Yangzhou University, Yangzhou 225009, China; 3 Key Laboratory of Plant Functional Genomics of the Ministry of Education, Jiangsu Key Laboratory of Crop Genomics and Molecular Breeding, Agricultural College, Yangzhou University, Yangzhou 225009, China

**Keywords:** leaf senescence, chlorophyll, grain filling, phytohormone

## Abstract

The mobilization and translocation of carbohydrates and mineral nutrients from vegetative plant parts to grains are pivotal for grain filling, often involving a whole plant senescence process. Loss of greenness is a hallmark of leaf senescence. However, the relationship between crop yield and senescence has been controversial for many years. Here, in this study, the overexpression and RNA interference lines of gene of *OsNYC3* (Non-Yellow Coloring 3), a chlorophyll catabolism gene, were investigated. Furthermore, exogenous phytohormones were applied, and a treatment of alternate wetting and moderate drying (AWMD) was introduced to regulate the processes of leaf senescence. The results indicated that the delayed senescence of the “STAY-GREEN” trait of rice is undesirable for the process of grain filling, and it would cause a lower ratio of grain filling and lower grain weight of inferior grains, because of unused assimilates in the stems and leaves. Through the overexpression of *OsNYC3*, application of exogenous chemicals of abscisic acid (ABA), and water management of AWMD, leaf photosynthesis was less influenced, a high ratio of carbohydrate assimilates was partitioned to grains other than leaves and stems as labeled by ^13^C, grain filling was improved, especially for inferior spikelets, and activities of starch-synthesizing enzymes were enhanced. However, application of ethephon not only accelerated leaf senescence, but also caused seed abortion and grain weight reduction. Thus, plant senescence needs to be finely adjusted in order to make a contribution to crop productivity.

## 1. Introduction

Rice (*Oryza sativa* L.) is the main source of calories for more than 2.5 billion people (http://faostat.fao.org/ accessed on 1 January 2021). Food production is supposed to increase by 70% to ensure world food security by year 2050 [1]. Thus, there is a great pressure to increase rice production. Rice production mainly depends on both the post-anthesis photosynthesis and the redistribution of prestored carbon reserves in vegetative organs. The redistribution of prestored carbon reserves could contribute up to 40% of the grain yield with an average value of 30% [2,3]. Due to the asynchronous growth and development of spikelets within a panicle, spikelets can be grouped into the superior and inferior ones. The superior spikelets located on apical primary branches flowered earlier in comparison to inferior spikelets on the proximal secondary branches. The superior ones usually fill fast and generate heavier grains, while inferior ones are either sterile or fill poorly [4,5]. Insufficient carbon partitioning from the source to the sink will result in poor grain filling in inferior spikelets, which limits rice grain yield [5].

The mobilization and translocation of carbohydrates and mineral nutrients from vegetative plant parts to grains often involve a whole-plant senescence process [6]. Plant senescence is regarded as a complex process in which multiple biochemical events happened parallelly or sequentially [7]. At certain developmental stages or triggered by environmental signals, the initiation of senescence starts with changes in the expression patterns of senescence-related genes. Such changes are not obvious at the very beginning of leaf senescence; they are soon easily visible as the degradation of chlorophyll pigments progresses [8]. These events are usually followed by the degradation of plastids, mitochondria, vacuoles, and nuclei, ending with cell death. Yellowing of leaves is a sign of senescence, which is related to the breakdown of chlorophyll. Chlorophyll, as a key component in leaf photosynthesis, is mostly assembled with thylakoid membrane proteins to form complexes such as the light-harvesting complexes and chlorophyll–protein complexes of photosystem I (PSI) and photosystem II (PSII), as well as the cytochrome *b_6_f* complex [9,10]. The breakdown of chlorophyll is a prerequisite to detoxify potentially phototoxic pigments within the vacuoles to permit the remobilization of nitrogen from the photosystem [11]. The chlorophyll metabolism pathways in rice involve chlorophyll biosynthesis genes such as *DVR*, *YGL8*, and *CAO1*, and catabolic genes such as *NYC3* and *SGR* [12,13,14].

Phytohormones including auxin, ethylene, abscisic acid (ABA), cytokinin (CK), gibberellic acid (GA), salicylic acid (SA), and brassinosteroids (BRs) implement their regulatory roles from early to later stages of senescence by integrating and synchronizing external signals with internal developmental processes [15,16,17,18]. The senescence process is facilitated by ethylene, ABA, SA, and BRs, whereas auxin, GA, and CKs contribute to retardation of senescence [17]. For example, ethylene is widely acknowledged as a senescence-promoting hormone both during ripening and under adverse environments [19]. ABA is a main developmental switch from functional anabolic to catabolic metabolism [20]. Cytokinins are known to retard senescence processes and even to induce regreening of yellowed leaves [21,22]. Many transgenic studies aimed at improving drought resistance and extending the photosynthetic active period have manipulated endogenous cytokinin biosynthesis through overexpression of the *IPT* gene under control of a senescence-inducible promoter [23]. Notably, water management of alternate wetting and moderate drying (AWMD) irrigation can modulate plant senescence by changing the content/ratio of endogenous hormones, e.g., ABA and ethylene levels [24,25].

The relationships between crop plant productivity and senescence have been disputed for many years [6]. Some studies have pointed out that the delayed senescence of “STAY-GREEN” contributes to higher production because of prolonged photosynthetic activity [26], but we hypothesize here that fine-tuned senescence through genetic modification, plant growth regulation, or crop management practices would accelerate the translocation of carbohydrate assimilates to grain, thus resulting in improved grain filling. Here, in this experiment, the *OsNYC3* (Non-Yellow Coloring 3) gene, encoding a plastid-localized α/β hydrolase-fold family protein involved in chlorophyll breakdown [27], was used by generating two *OsNYC3* overexpression (9522-OX) and two RNA interference (9522-RI) transgenic lines in a rice cultivar 9522. The exogenous phytohormones were applied, and treatment of AWMD was introduced to investigate the relationships between plant senescence and grain filling in rice, along with the regulatory role of phytohormones.

## 2. Results

### 2.1. Yield and Yield Components

Regulating the expression level of *OsNYC3* had a significant effect on yield and yield components (Table 1). Grain yields were on average 7.02% higher in 9522-OX lines than the wildtype. The grain yields of 9522-RI lines were on average 2.64% lower than the wildtype, but the difference was not significant. The differences in grain yields were mainly due to the increased percentage of filled grains and heavier 1000-seed weight in 9522-OX lines, while the opposite results were observed in 9522-RI lines. The application of exogenous phytohormones on rice leaves and water management of AWMD also significantly influenced grain yields and yield components, with the highest yield in the treatment of AWMD, followed by treatments of ABA, AVG, 6-BA, and ETH. For yield components, the number of spikelets per panicle, percentage of filled grains, and 1000-seed weight were mostly affected by different treatments, which resulted in the differences in grain yields.

### 2.2. Leaf Photosynthesis-Related Traits

**Figure 1:**
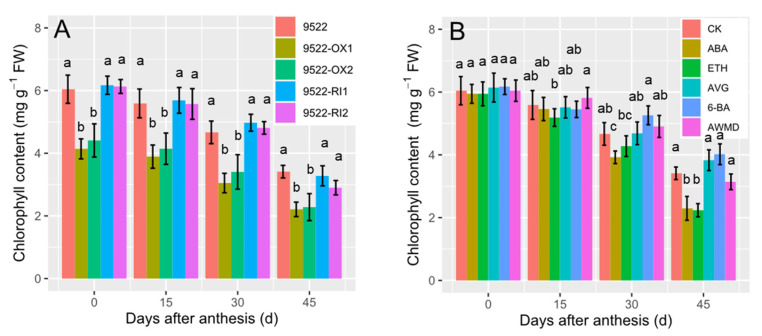
Changes in chlorophyll contents of flag leaves of wildtype and transgenic lines (**A**), and application of exogenous phytohormone/alternate wetting and under moderate drying (AWMD) irrigation (**B**) during grain filling (0–45 days after anthesis). 9522-OX1 and 9522-OX2, two transgenic lines with overexpression of *NYC3* (Non-Yellow Coloring 3) in variety 9522; 9522-RI1 and 9522-RI2, two RNA interference transgenic lines in variety 9522; ABA, exogenous application of abscisic acid; ETH, exogenous application of ethephon (an ethylene-releasing agent); AVG, exogenous application of aminoethoxyvinylglycine (an inhibitor of ethylene synthesis by inhibiting 1-aminocylopropane-1-carboxylic acid synthesis); 6-BA, exogenous application of benzylaminopurine (a synthetic cytokinin). Different letters above columns indicate statistical significance at the *p* < 0.05 level between different genetic lines/treatments of each plant organ. Values are the means ± SD.

**Figure 2:**
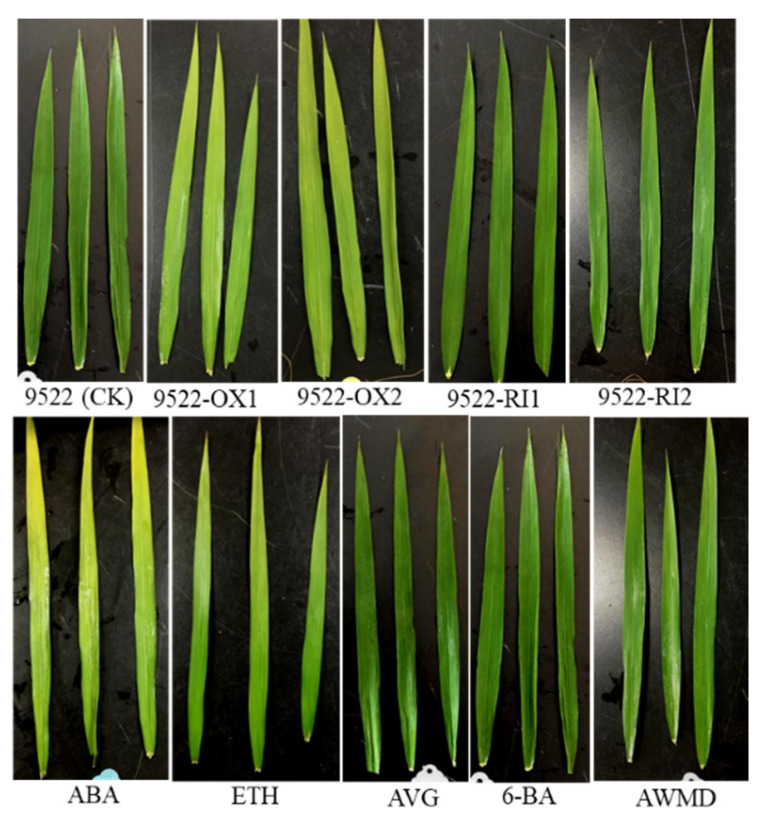
Changes in leaf color of wildtype and transgenic lines, following application of application of exogenous phytohormone/alternate wetting and under moderate drying (AWMD) irrigation at 15 days post anthesis. Symbols are as described in Figure 1.

The *OsNYC3* gene, encoding a plastid-localized protein with an esterase/lipase motif, is involved in chlorophyll degradation. Generally, leaf chlorophyll content decreased gradually after anthesis (Figure 1). When comparing chlorophyll content between wildtype and transgenic lines, the value is 30% lower in the 9522-OX line than in the wildtype, but there were no significant differences between them (Figure 1 and Figure 2). Rice leaves responded differently to exogenous phytohormones or water treatment of AWMD. ABA and ETH accelerated the process of leaf senescence and resulted in lower chlorophyll content. Comparatively, water management of AWMD also reduced chlorophyll content, but to a lesser extent. On the other hand, the treatments of AVG and 6-BA retained a higher chlorophyll content in leaves than the control treatment, even at the late grain filling stage.

**Figure 3:**
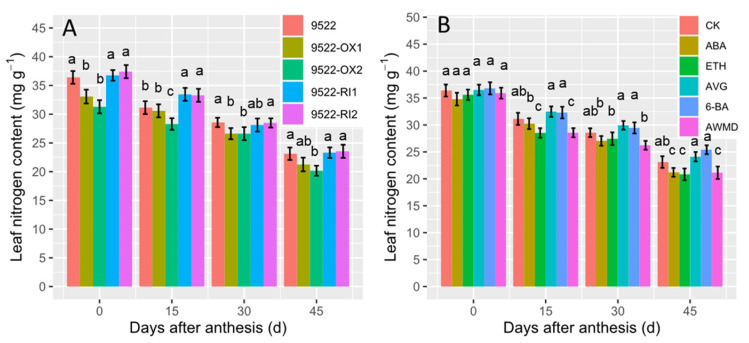
Changes in flag leaf nitrogen contents of wildtype and transgenic lines (**A**), and application of exogenous phytohormone/alternate wetting and under moderate drying (AWMD) irrigation (**B**) during grain filling (0–45 days after anthesis). Symbols and significance levels are as described in Figure 1.

Leaf nitrogen is very important for photosynthetic carbohydrate assimilates, as enough nitrogen is needed to build photosystems and related enzymes. The change in leaf nitrogen content with time during grain filling was also examined (Figure 3). After anthesis, leaf nitrogen content decreased gradually. However, leaf nitrogen was lower in 9522-OX lines than in 9522-RI lines. Leaf nitrogen content were similar among different treatments at beginning of grain filling, but a prominent decrease was observed in the treatments with ABA, ETH, and AWMD. Meanwhile, application of AVG and 6-BA increased leaf nitrogen content by 3.18% and 3.97%, respectively, when compared with control plants.

**Figure 4:**
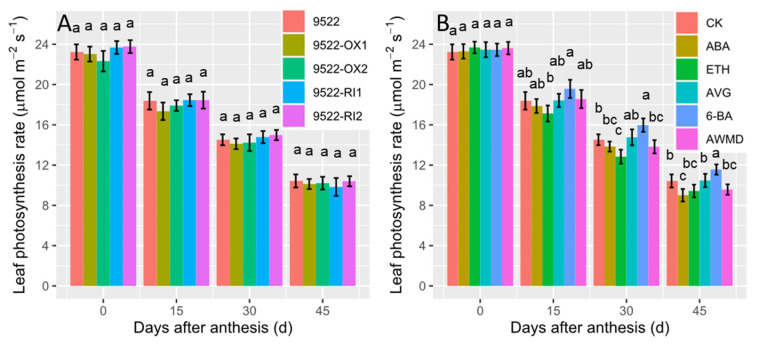
Changes in flag leaf photosynthesis rates of wildtype and transgenic lines (**A**), and application of exogenous phytohormone/alternate wetting and under moderate drying (AWMD) irrigation (**B**) during grain filling (0–45 days after anthesis). Symbols and significance levels are as described in Figure 1.

Leaf photosynthesis is pivotal for providing carbohydrate assimilates for the grain filling process. In Figure 4, there were no significant differences in photosynthesis rate between wildtype and transgenic lines. However, following application of exogenous phytohormone, leaf photosynthesis was higher in treatments of AVG and 6-BA but lower in treatments of ABA and ETH, when compared with control plants. Photosynthesis rate was also decreased in treatment of AWMD, especially at the late grain filling stage, which may be related to the translocation of photosynthetic assimilates from stems and leaves to grains. The changes in photosynthesis rate were parallelized to the changes in leaf nitrogen content (Figure 3), as most nitrogen is used to construct photosynthetic system.

**Figure 5:**
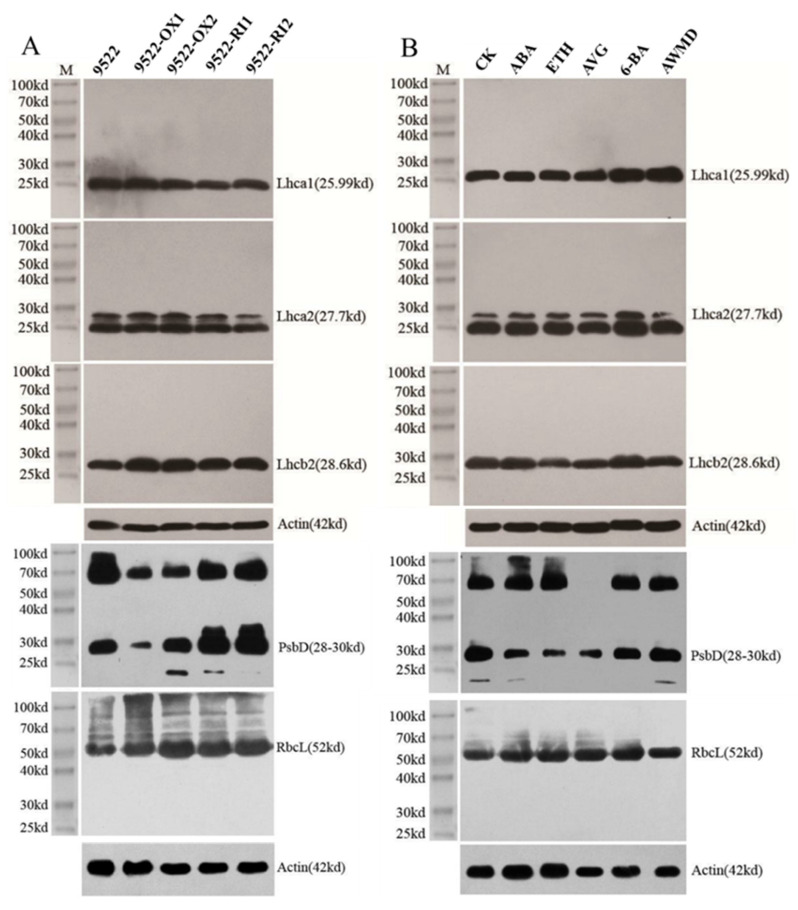
Western blot analysis of photosynthetic proteins from leaves of wildtype and transgenic lines at heading stage (**A**), and 15 days after the application of exogenous phytohormone/alternate wetting and under moderate drying (AWMD) irrigation (**B**). Lhca1, Lhca2 are light-harvesting complex I (LHCI) apoproteins. Lhcb2 is an LHCII apoprotein. PsbD is the D2 reaction center polypeptide of photosystem II. RbcL is the large subunit of Rubisco. Symbols are as described in Figure 1.

To examine the contents of photosynthetic proteins in detail, Western blot analysis was performed (Figure 5). There were no significant differences in the major LHCI apoproteins of Lhca1 and Lhca2 and the major LHCII apoprotein of Lhcb2 between wildtype and transgenic lines. PsbD, a D2 reaction center polypeptide of photosystem II, was degraded in 9522-OX lines; however, reduced degradation was observed in 9522-RI lines. RbcL, the large subunit of Rubisco, was not influenced by the expression levels of *OsNYC3*, which is consistent with the photosynthesis rate during the grain filling stage (Figure 4). Application of 6-BA significantly increased the contents of proteins of Lhca1 and Lhca2, which indicated that the sizes of light harvest complexes in PSI and PSII were increased, and a similar result was also observed in the treatment of AWMD at 15 days post anthesis. For the reaction center of photosystem II, the protein contents of PsbD were significantly decreased in the treatments with ABA and ETH, which may be related to their roles in leaf senescence promotion. Rubisco content was also improved by the treatment with 6-BA compared to CK, which is consistent with the high photosynthesis rate of 6-BA treatment (Figure 4).

### 2.3. Grain Filling and Redistribution of Assimilates from Carbon Reserves in Stems

**Figure 6:**
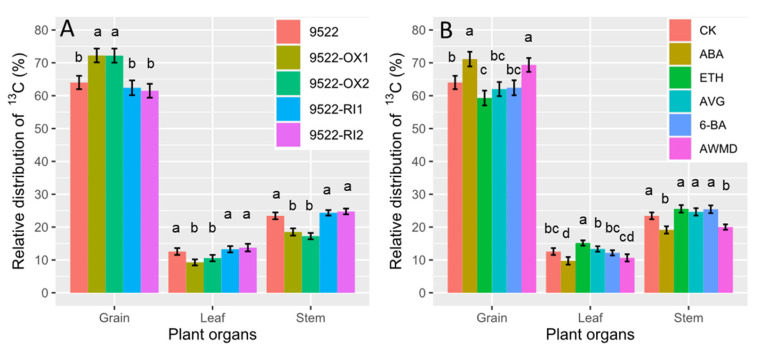
Relative partitioning ^13^C in the grains, leaves, and stems of wildtype and transgenic lines (**A**), and application of exogenous phytohormone/alternate wetting and under moderate drying (AWMD) irrigation (**B**) at harvest stage. Symbols and significance levels are as described in Figure 1.

The comparative study of ^13^C translocation from leaves to shoots and grains showed that overexpression of *OsNYC3* would favor the partition of photosynthetic assimilates to grains (Figure 6). As shown in Figure 5, 12.8% more but 23.7% less carbohydrates labeled at anthesis were later translocated to grains and stems, respectively, resulting in 9.9% less unused carbohydrates in leaves, when comparing 9522-OX lines with wildtype plants. In contrast, no significant differences were found between 9522-RI lines and the wildtype. Treatment with ETH, AVG, and 6-BA retained ^13^C-labeled carbohydrates in either leaves or stems, resulting in a low translocation ratio to grains, which is adverse for grain filling. The relative distribution of ^13^C to grains was only higher than control plants in treatments with ABA and AWMD.

**Figure 7:**
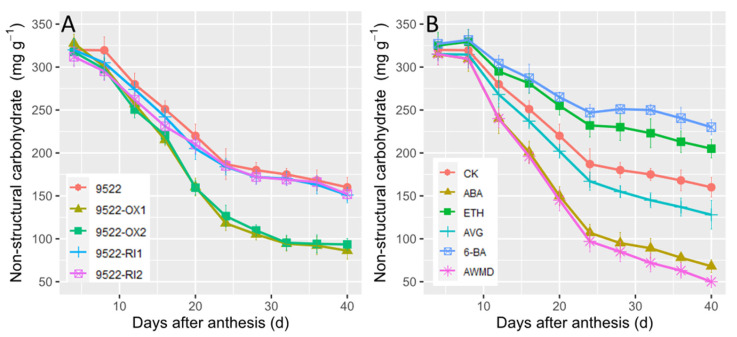
Changes in contents of nonstructural carbohydrates (NSCs) in the stems of wildtype and transgenic lines (**A**), and application of exogenous phytohormone/alternate wetting and under moderate drying (AWMD) irrigation (**B**) during grain filling stage. Symbols and significance levels are as described in Figure 1.

The total sugars as nonstructural carbohydrates (NSCs, including soluble sugars + starch) in stems decreased during grain filling (Figure 7). The decrease in NSC was faster in 9522-OX lines than in 9522-RI lines and wildtype plants. NSC contents sharply decreased from 8 to 28 days after anthesis, and overexpression of *OsNYC3* accelerated this process. The changes in NSC concentration varied across different treatments, with ABA and AWMD causing the sharpest decreases in NSC of the stems, but 6-BA and ETH impeded the progress of reallocation of pre-anthesis assimilates from the stems to grains. These results were consistent with the ^13^CO_2_ labeling data (Figure 6).

**Figure 8:**
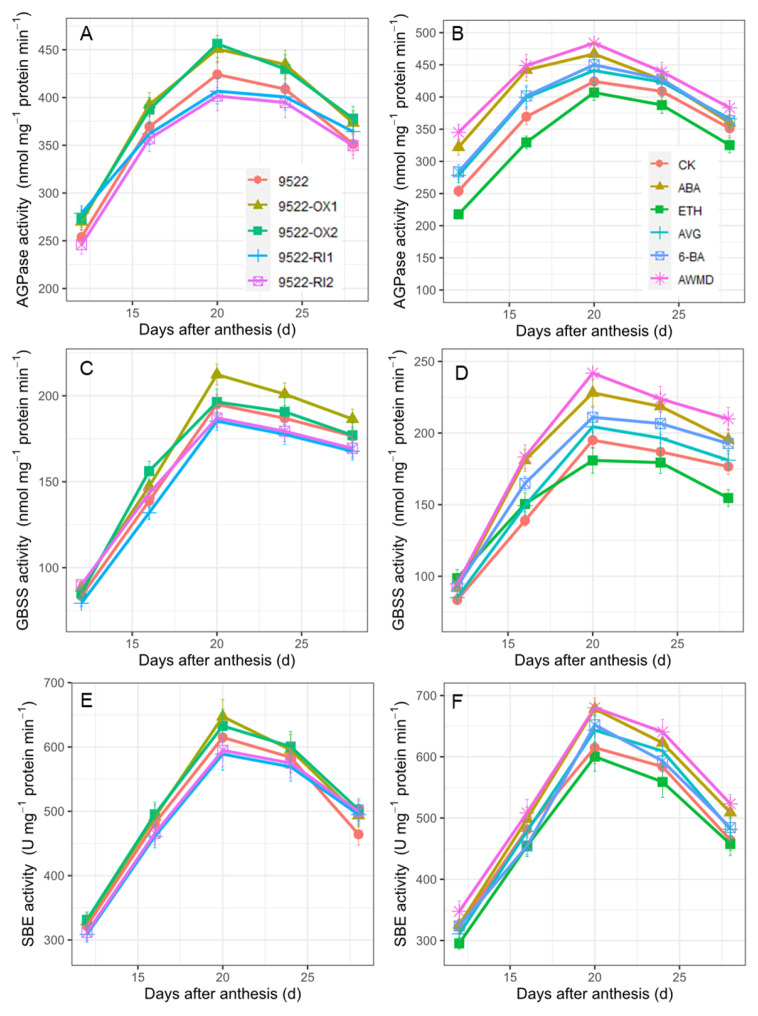
Activities of adenosine diphosphate-glucose pyrophosphorylase (AGPase; **A**,**B**), granule-bound starch synthase (GBSS; **C**,**D**), and starch branching enzyme (SBE; **E**,**F**) in spikelets of rice of wildtype and transgenic lines (**A**,**C**,**E**), and application of exogenous phytohormone/alternate wetting and under moderate drying (AWMD) irrigation (**B**,**D**,**F**) during grain filling stage. Symbols and significance levels are as described in Figure 1.

Changes in the activities of key enzymes in starch synthesis of adenosine diphosphate–glucose pyrophosphorylase (AGPase), granule-bound starch synthase (GBSS), and starch branching enzyme (SBE) were very similar (Figure 8). Activities of these enzymes reached their peak around 20 days after anthesis, and then gradually decreased at the later stage of grain filling. The activities of AGPase, GBSS, and SBE were enhanced in 9522-OX lines, but decreased in 9522-RI lines. Treatments of AWMD, ABA, and AVG also increased the activities of starch synthesis-related enzymes, whereas their activities were lower following ETH treatment.

**Figure 9:**
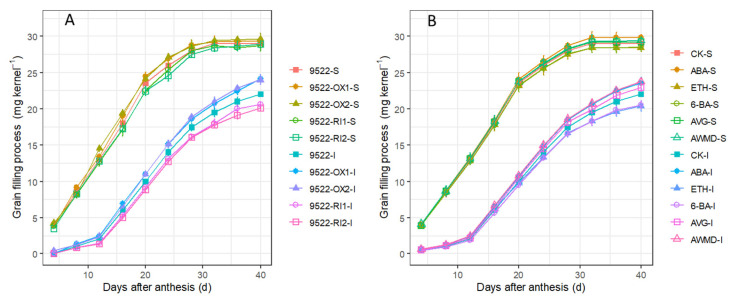
Grain-filling processes of superior (S) and inferior (I) spikelets of wildtype and transgenic lines (**A**), and application of exogenous phytohormone/alternate wetting and under moderate drying (AWMD) irrigation (**B**) at harvest stage. Symbols and significance levels are as described in Figure 1.

The grain filling rate and grain weight were much greater for superior spikelets than for inferior spikelets (Figure 9). They were substantially increased by overexpression of *OsNYC3* for inferior spikelets when compared with those of the wildtype, but showed no significant difference between the wildtype and transgenic lines for superior spikelets. The application of exogenous phytohormone or water management of AWMD also impacted grain filling rate and grain weight. AWMD and ABA improved the grain filling of inferior spikelets, while 6-BA and ETH impeded the grain filling of inferior spikelets. As a result, grain yield was increased by 9.0% and 5.7% following the treatments of AWMD and ABA, respectively, while it was reduced by 2.1% under the ETH treatment, relative to that under the control environment (Table 1).

## 3. Discussion

### 3.1. The Relationships between “STAY-GREEN” Trait and Crop Production

Senescence is a highly programmed recycling process in which carbohydrates and mineral nutrients are remobilized from deteriorating leaves to developing grains [28]. The hallmark of senescence is yellowing of leaves because of preferential degradation of chlorophyll over carotenoids. Mutants with the “STAY-GREEN” phenotype are reported in many species, and the most extensively studied are the nonfunctional/cosmetic “STAY-GREEN” mutants which exhibit loss of function of photosynthetic capacity, but retain leaf greenness [29]. This is also the case for 9522-RI lines, in which chlorophyll catabolism is inhibited (Figure 1), but photosynthetic capacity is similar to the wildtype (Figure 4). In this case, “STAY-GREEN” is defined as unfavorably delayed because translocation of nutrients from leaves to newly developed seeds was impeded and unused assimilates were left in the straw (Figure 6 and Figure 7). In 9522-OX lines, early leaf senescence is accompanied by improved grain filling, resulting in a high grain filling rate and grain weight (Table 1).

Several studies on different crops, genotypes, and transgenic lines have indicated that cultivars with delayed senescence and prolonged photosynthetic activity, which are characteristics of functional “STAY-GREEN” plants, could increase productivity [6,26,28]. Cytokinins have long been recognized to retard senescence processes, and most transgenic approaches aiming at a functional “STAY-GREEN” trait improve the endogenous cytokinin content through the expression of the *IPT* gene (a key gene in cytokinin biosynthesis) under control of a senescence-inducible promoter *SAG12*. This strategy was first successfully demonstrated in tobacco [30]. Tobacco *P_SAG12_::IPT* transformants significantly delayed the senescence of lower leaves, increased biomass weight by 40%, and increased seed yield by 52% [30]. This successful attempt in tobacco plants prompted many researchers to transfer the *IPT* gene to extend the photosynthetic active period in many agronomically important crops [31,32,33]. The effect of the IPT gene on improving or stabilizing crop yields is especially apparent under abiotic stress environments; for example, by controlling the IPT gene expression of a stress- and maturation-induced promoter of a gene encoding a receptor protein kinase (SARK), the “STAY-GREEN” transgenic lines retained leaf water contents, maintained a high photosynthesis rate, and showed extreme drought tolerance [23]. Moreover, the transgenic plants saved as much as 70% of irrigation water with minimal yield loss.

However, this relationship is more complicated for cereal crops regarding grain yield. There has long been a dispute on whether yield is determined by sink sizes or by source activities. The predominant current conclusion is that sink size is the primary determinant of yields, especially for cereal crops with small size grains [34]. Therefore, the “STAY-GREEN” trait is sometimes defined as unfavorably delayed because the gain from the extended period of photosynthesis is less than the loss due to incomplete remobilization of prestored carbohydrates in the straw [5]. For example, the “STAY-GREEN” trait in the late reproductive stage of wheat by the expression of the *IPT* gene has no positive effect on yield [35]. It is proposed that the “STAY-GREEN” trait interferes with the wheat reproductive strategy based on fast senescence and remobilization of metabolites from senescing organs to developing grains shortly after anthesis [35]. Similar results were observed in rice [36,37]. These results are consistent with our study in which overexpression of *OsNYC3* (a regulator of chlorophyll degradation) accelerated leaf senescence (Figure 1 and Figure 2), while translocation of NSC in the stem contributed to improved grain filling rate and seed weight, especially inferior ones (Figure 7 and Figure 8, Table 1).

### 3.2. Leaf Senescence Needs to Be Finely Tuned to Increase Yields for Cereal Crops

There are reports illustrating the negative correlations between the “STAY-GREEN” trait at the late reproductive stage and grain yield in cereal crops. For example, in a double-haploid population of rice derived from a cross of an *indica* parent and a “STAY-GREEN” *japonica* parent, the “STAY-GREEN” trait was associated with yield losses [38]. Naruoka et al. [39] also reported a negative correlation of yield with the “STAY-GREEN” characteristic in wheat lines under wet, cool conditions. Poor grain filling is the main limitation for cereal plants to achieve their yield potential; it is always associated with delayed senescence and results in lower grain yield [40,41]. Generally, the overuse of nitrogen fertilizers [42], the introduction of lodging-resistant cultivars that stay green for too long [43], and delayed whole-plant senescence of hybrids [44] were the main reasons for the unfavorably delayed senescence. In these cases, a high amount of prestored NSCs are left unused in stems and plants that remain green, whereas grains are due to ripen [45,46].

Thus, regulation of senescence needs to be finely turned to increase grain yield [46]. Senescence can also be altered by senescence-promoting or -inhibiting hormones. ABA is not only well known for the rapid response to external stimulus, e.g., hot and drought stress [47], but also involved in the regulation of numerous internal processes of plant development, such as seed germination, root development, senescence, and seed development [47,48]. Although it has been reported that high ABA levels suppress starch synthesis in maize [49] and reduce the grain weight of wheat [50], evidence has shown that a low dose of ABA results in increases in grain filling rate and grain weight in different crops [51,52]. As shown in Figure 7, the translocation of NSCs was significantly improved following the ABA treatment. Ethylene is a major hormone hastening leaf senescence, fruit ripening, and flower senescence [7,15]. Leaf senescence is modulated by the application of ETH and AVG (Figure 1). However, overproduction of ethylene is frequently related to fruit abortion and grain weight reduction [53,54], which is consistent with the lower weight of inferior grains following ETH treatment (Figure 9, Table 1). Cytokinins regulate the proliferation and differentiation of plant cells [21]. Following application of 6-BA to the leaves, leaf senescence was impeded and unfavorably delayed, which resulted in poor grain filling in this study (Figure 9). Notably, water management of AWMD significantly improved grain filling of grains, especially inferior ones (Table 1, Figure 9). AWMD could significantly increase ABA synthesis in grains, especially inferior ones, reduce both the ethylene release rate and the concentration of 1-aminocyclopropane-1-carboxylic acid (ACC, the precursor of ethylene), and increase the ratio of ABA to ethylene, thereby enhancing grain filling and grain weight, especially in inferior grains [24,55,56,57,58,59]. The changes in phytohormone levels could result in enhanced activity and/or gene expression of enzymes involved in sucrose-to-starch conversion (Figure 8) [60].

The process of grain filling is pivotal for rice production, and it overlaps with plant senescence, but their relationship remains elusive. Through manipulation of the expression levels of a chlorophyll catabolism gene, *OsNYC3*, application of exogenous chemicals, and water management of AWMD, we found that the leaf “STAY-GREEN” trait is unfavorable, because rice grain production is largely based on fast senescence and remobilization of metabolites from senescing organs to grains shortly after heading. Leaf/plant senescence needs to be finely tuned to increase grain yields. We showed that manipulation of the expression levels of senescence-related genes, application of exogenous chemicals of abscisic acid (ABA), and water management of AWMD could be an alternative approach to improve the grain filling of rice.

## 4. Materials and Methods

### 4.1. Plant Materials

A japonica cultivar 9522 was selected as the wildtype. For the generation of 9522-RI, the partial exons 1 and 2 containing *OsNYC3* were amplified from *japonica* rice Nipponbare cDNA using primers RI-F (5′-AAAGGATCCACAATAGCAAGGCACCG-3′) and RI-R (5′-AAAACTAGTA GCGAGGAGATGTAGCAG-3′). The target fragment was then cloned into the p1022 vector and transferred into the plant binary vector *p1301UbiNOS*, expressed under the control of the maize *ubiquitin* promoter. To generate the 9522-OX construct, the coding region of *OsNYC3* was amplified from Nipponbare cDNA using OX-F (5′-AAAACTAGTATGGAAGTGGTTTCTTCCAGCCACTC-3′) and OX-R (5′-AAAGAGCTCTTATCTAGATATTACCCATGTGTTGGA-3′), inserted into the *p1301UbiNOS* vector. All constructs were transformed via *Agrobacterium tumefaciens*-mediated transformation.

### 4.2. Plant Growth Environment and Treatment

The wildtype (9522), 9522-RI, and 9522-OX lines were grown in fields at the experimental station of Yangzhou University, Jiangsu Province, China. For each genetic line, there were three replicates/plots. Each plot occupied 30 m^2^ (5 m in width and 6 m in length). The distances between plants and between rows were 13.3 and 25 cm, respectively. The water management maintained a continuous flood with 2–3 cm water depth until 1 week before the final harvest. Weeds, insects, and diseases were controlled using chemical or manual methods to avoid yield loss. Phosphorus (30 kg·ha^−1^ as single superphosphate) and potassium (40 kg·ha^−1^ as KCl) were applied and incorporated as basal fertilizer before transplanting. Furthermore, 170 kg N·ha^−1^ was applied in a ratio of 5:2:3 at the pre-transplanting, early tillering, and panicle initiation stages.

For genotype 9522, starting at 6 days after full heading, ABA, ethephon (ETH), aminoethoxyvinylglycine (AVG), and 6-benzylaminopurine (6-BA) were applied to the leaves; from full heading, the water treatment of AWMD was introduced. The preparation and application of the chemical solutions were as described elsewhere [24,61]. In brief, starting at 6 days after full heading, 20 μmol·L^−1^ ABA, 50 mmol·L^−1^ ethephon (ETH, an ethylene-releasing agent), 50 μmol·L^−1^ aminoethoxyvinylglycine (AVG; an inhibitor of ethylene synthesis by inhibiting ACC synthesis), and 150 μmol·L^−1^ 6-benzylaminopurine (6-BA, a synthetic cytokinin) were applied to the top three leaves using a writing brush which was dipped in the solutions. The chemicals were applied daily for 4 days at the rate of 4 mL per panicle at each application, with 0.5% (*v*/*v*) Teepol (Fluka, Riedel-de-Haen, Seelze, Germany) as a surfactant. The same volume of deionized water containing the same concentrations of Teepol was applied to the control plants. For water management, the irrigation regime of AWMD was introduced, in which, from heading to maturity, fields were not irrigated until the soil water potential reached −15 kPa at 15–20 cm depth.

### 4.3. SDS-PAGE and Protein Gel Blot Analysis

Flag leaves of the wildtype and transgenic lines at the heading stage and 15 days after the application of exogenous phytohormone/AWMD treatment were used for protein extraction and Western blot analysis. Then, 100 mg (fresh weight) of leaf samples were ground in liquid nitrogen, and total leaf protein was extracted using 5 mL of extraction buffer (62.5 mM Tris, pH 6.8, 2.5% SDS, 5% mercaptoethanol, 10% glycerol). The extracted leaf proteins were denatured by adding SDS-PAGE loading buffer at 100 °C for 5 min before SDS-PAGE analysis. The samples were isolated in 12.5% SDS polyacrylamide gel and blotted on polyvinylidene fluoride (PVDF) transfer membranes (Merck Millipore, Burlington, MA, USA). Specific antibodies were incubated with the membranes, and the target proteins were detected using an eECL Plus Western Blotting Detection System (GE Healthcare UK Ltd., Amersham, UK). Protein images were obtained using a Tanon-5200 instrument (Shanghai, China). Antibodies against Lhca1, Lhca2, Lhcb2, PsbD, and RbcL were purchased from Agrisera (http://www.Agrisera.com/ accessed on 1 January 2021).

### 4.4. ^13^CO_2_ Labeling and Sampling

At the flowering stage, 10 plants from each genetic line/treatment were labeled with ^13^CO_2_. Flag leaves of the main stems were used for isotope labeling under ambient field conditions, which lasted for 30 min, between 9:00 a.m. and 11:00 a.m., on a clear day with photosynthetic active radiation at the top of the canopy ranging between 1000 and 1400 μmol·m^−2^·s^−1^. The whole flag leaf was individually enclosed in a polyethylene bag (25 cm length and 4 cm diameter) and exposed to a ^13^CO_2_-enriched atmosphere. ^13^C was supplied by the injection of 15 mL of ^13^CO_2_ gas (from Shanghai Research Institute of Chemical Industry Co., Ltd., Shanghai, China). At the maturity stage, the labeled plants were harvested, and each plant was divided into leaf blades, culms plus sheaths, and kernels. To determine total C and δ^13^C, samples were combusted in an elemental analyzer (CHNS elemental analyzer, Vario marco cube, Elementar, Germany) and an isotope ratio mass spectrometer (Isoprime100, Elementar, Germany). Isotopic values are expressed in d units, denoting parts per thousand deviations (‰) relative to VPDB (Vienna Pee Dee Belemnite).

### 4.5. NSC Measurement, Extraction and Assays of Starch Metabolic Enzymes

The method for extraction of NSC in stems was as described earlier [62]. Eight to ten tagged panicles were sampled from each plot at 12, 16, 20, 24, and 28 days after anthesis. Spikelets were removed from the tagged panicles, frozen in liquid nitrogen for 2 min, and then stored at −70 °C for the measurement of enzymatic activity. All chemicals and enzymes used for enzymatic measurement were from Sigma Chemical Company (St. Louis, MO, USA). All enzyme assays were optimized for pH and substrate concentration, and they were within the linear phase with respect to incubation time and protein concentration. Protein content was determined according to Bradford [63], using bovine serum albumin as the standard. The extraction procedure for AGPase, GBSS, and SBE was according to Nakamura et al. [64]. The enzyme activities were determined as described previously for AGPase, SBE [64], and GBSS [65]. Activities of enzymes were expressed as U·mg^−1^ protein·min^−1^ for SBE and nmol·mg^−1^ protein·min^−1^ for the others.

### 4.6. Statistical Analysis

Analysis of variance was performed using R programming language [66]. The least significant difference (LSD) test was conducted for multiple comparisons between genetic lines or treatments. For figures, the data were visualized using the ggplot 2 package [67].

## Figures and Tables

**Table 1:** The effects of gene Os*NYC3* (a regulator of chlorophyll degradation), application of exogenous phytohormone, and alternate wetting and moderate drying (AWMD) irrigation on grain yield and yield components.

Genetic Materials	Treatment	Number of Panicles per m^2^	Number of Spikelets per Panicle	Percentage of Filled Grains (%)	1000-Seed Weight (g)	Yield (t·ha^−1^)
9522	CK	243.33 ± 4.98 ^b^	133.53 ± 3.51 ^ab^	81.33 ± 1.25 ^b^	27.2 ± 0.15 ^c^	7.19 ± 0.19 ^b^
9522-OX1	CK	238.76 ± 5.17 ^b^	136.70 ± 3.45 ^ab^	83.60 ± 2.35 ^ab^	28.3 ± 0.27 ^ab^	7.72 ± 0.24 ^a^
9522-OX2	CK	243.57 ± 4.05 ^b^	130.78 ± 4.27 ^b^	84.50 ± 1.57 ^a^	28.5 ± 0.25 ^a^	7.67 ± 0.15 ^a^
9522-RI1	CK	255.22 ± 5.12 ^a^	142.25 ± 5.12 ^a^	72.53 ± 2.15 ^d^	26.5 ± 0.12 ^d^	6.98 ± 0.21 ^b^
9522-RI2	CK	254.35 ± 4.65 ^a^	142.64 ± 4.15 ^a^	72.52 ± 1.51 ^d^	26.7 ± 0.26 ^d^	7.02 ± 0.22 ^b^
9522	ABA	240.27 ± 6.87 ^b^	137.21 ± 4.25 ^ab^	82.37 ± 2.12 ^ab^	28.0 ± 0.12 ^b^	7.60 ± 0.19 ^a^
9522	ETH	245.41 ± 4.00 ^b^	140.08 ± 3.25 ^a^	78.20 ± 1.52 ^c^	26.2 ± 0.11 ^e^	7.04 ± 0.25 ^b^
9522	6-BA	241.96 ± 5.34 ^b^	135.42 ± 4.42 ^ab^	81.53 ± 2.14 ^b^	27.0 ± 0.24 ^c^	7.22 ± 0.2 ^b^
9522	AVG	242.45 ± 7.02 ^b^	136.00 ± 5.55 ^ab^	80.35 ± 1.04 ^bc^	27.3 ± 0.22 ^c^	7.24 ± 0.14 ^b^
9522	AWMD	238.63 ± 6.56 ^b^	141.20 ± 4.12 ^a^	82.71 ± 2.71 ^ab^	28.1 ± 0.29 ^ab^	7.84 ± 0.27 ^a^

Different letters indicate statistical significance at the *p* = 0.05 level with the same column.

## Data Availability

Not applicable.
